# The influence of neuropsychiatric and functional changes on quality of life in Alzheimer's disease

**DOI:** 10.1590/S1980-57642010DN40100008

**Published:** 2010

**Authors:** Marcia M.P.C. Novelli, Paulo Caramelli

**Affiliations:** 1 Occupational Therapy Course, Department of Health Sciences, Federal University of São Paulo, Santos SP, Brazil.; 2 Behavioral and Cognitive Neurology Unit, Department of Neurology, University of São Paulo School of Medicine, São Paulo SP, Brazil.; 3 Behavioral and Cognitive Neurology Unit, Department of Internal Medicine, Faculty of Medicine, Federal University of Minas Gerais, Belo Horizonte MG, Brazil.

**Keywords:** quality of life, Alzheimer’s disease, neuropsychiatric symptoms, functional performance, caregivers

## Abstract

To investigate the influence of neuropsychiatric manifestations and functional performance on quality of life (QOL) of AD patients and their caregivers/family members.

**Methods:**

The QOL-AD scale, Mini-Mental State Examination (MMSE), Geriatric Depression Scale (GDS), Cornell and Beck Scales for Depression, Physical and Instrumental-Self Maintenance scales (AIDL and ADL) and the Neuropsychiatric Inventory (NPI) were applied to 60 patients with probable AD, mild (n=30) or moderate (n=30) dementia, according to NINCDS-ADRDA and DSM-III-R criteria, respectively, and to their caregivers/family members. The total scores on the three QOL-AD versions were correlated with the measures previously mentioned.

**Results:**

The QOL-AD patients' version displayed significant correlations with GDS (–0.76 p<0.01), Cornell (–0.53 p<0.01) and NPI (–0.46 p<0.05) in the mild dementia subgroup. The caregivers' version about patients' QOL correlated with GDS (–0.48 p<0.01), Cornell (–0.57 p<0.01), NPI (–0.46 p<0.01) and AIDL (–0.36 p<0.05), while the caregivers' version about their own QOL was significantly correlated with NPI (–0.43 p<0.01), AIDL (–0.35 p<0.05) and Beck Depression (–0.67 p<0.01). In the moderate dementia subgroup, significant correlations were observed with GDS (–0.45 p<0.05) and Cornell (–0.46 p<0.01). For the caregivers' version about patients' QOL, significant correlations emerged with Cornell (–0.68 p<0.01), NPI (–0.67 p<0.01), AIDL (–0.41 p<0.05), ADL (–0.49, p<0.01) and Beck Depression (–0.33 p<0.05). For the caregivers' version about their own QOL, significant correlations with Beck Depression (–0.54 p<0.01) and ADL (–0.38, p<0.05) were found.

**Conclusion:**

The symptoms presented in AD affected the QOL in patients and caregivers/family members in both mild and moderate dementia.

## Abstract (pt)

Investigar avaliar a influência das manifestações neuropsiquiátricas e do desempenho funcional sobre a qualidade de vida dos pacientes com DA e de seus cuidadores/familiares.

**Métodos:**

A escala QdV-DA, Mini-exame do Estado Mental (MEM), Escala de Depressão Geriátrica (GDS), Inventário de Depressão de Beck e a Escala de Cornell para depressão, Escala de atividades de vida diária (ABVD e AIVD) de Lawton e Brody e o Inventário Neuropsiquiátrico (INP) foram administrados em uma amostra de 60 pacientes com DA provável, segundo os critérios do NINCDS-ADRDA , com gravidade de leve (n=30) a moderado (n-30) segundo os critérios do DMS-III-R. e a seus respectivos cuidadores/familiares. A pontuação total, das três versões da escala, foi correlacionada aos instrumentos previamente mencionados.

**Resultados:**

A versão de QV dos pacientes no grupo de demência leve apresentou correlações significativas com o GDS (–0,76 p<0,01), Cornell (–0,53 p<0,01) e INP (–0,46 p<0, 05). A versão do cuidador sobre a QV do paciente se correlacionou com: GDS (–0,48 p<0,01), Cornell (–0,57 p<0,01), INP (–0,62 p<0,01) e AIVD (–0,36 p<0,05) enquanto que a versão do cuidador sobre sua própria QV se correlacionou significativamente com NPI (–0,43 p<0,01), AIVD (–0,35 p<0,05) e GDS (–0,67 p<0,01). No grupo de pacientes moderados foram encontradas correlações significativas com GDS (–0,45 p<0,05) e a Escala de Cornell (–0,46 p<0,01). Para versão do cuidador sobre a QV do paciente as correlações significativas foram com: Cornell (-0,68 p<0,01), INP (-0,67 p<0,01), AIVD (-0,41 p<0,05), ABVD (-0,49 p<0,01), e Inventário de Depressão de Beck (-0,33 p<0,05). Para versão do cuidador sobre sua própria QV foram encontradas correlações significativas com: Inventário de Depressão Beck (-0,54 p<0,01) e ABVD (-0,38 p<0,05).

**Conclusão:**

Os sintomas da DA afetam a QV dos pacientes como também dos cuidadores/familiares tanto na fase leve como na fase moderada.

The construct of quality of life (QOL) and its use as an outcome measure have been widely studied in chronic degenerative diseases, including dementia. QOL is considered a multidimensional construct and the most used definition of QOL in dementia was proposed by Whitehouse and Rabins,^1^ where it is considered a combination of cognitive functioning, activities of daily living, social interaction and well-being.

Alzheimer's disease (AD) is a clinical condition characterized by cognitive decline leading to significant impairment in patients' daily life activities, social and occupational performance^2^ and which significantly interferes with patients’ and caregivers' QOL.^1,3-6^

The QOL studies recently published have focused on the proposal of reliable and valid instruments for these assessments. However, few studies have attempted to identify the influence of the symptoms of the disease on quality of life in both patients and caregivers/family members.

The few studies conducted to date have identified that the QOL of patients is influenced by cognitive impairment^3^, depressive symptoms reported by patients,^3,5.7-10^ functional performance, considering instrumental (IADL) and basic (BADL) activities of daily living,^3,5.8,10^ behavioral disturbances^3,5.7,11,12^ and patients’ depressive symptoms reported by caregivers.^3,5.10^

Most of these studies evaluated the influence of these variables in isolation and based their analysis on patients' self-reports. However, a recently published study^13^ identified the influence of anosognosia on self-report, an important feature to take into account in dementia.

One way to minimize the influence of anosognosia on QOL in evaluations is to consider the proxy report as an additional source of information. This kind of evaluation was only considered in the study by Logsdon and co-workers.^3^

Studies with caregivers/family members have not been frequently conducted. One study by Vellone, et al.^14^ reported that caregivers associated good QOL with serenity, tranquility, psychological well-being, freedom, general well-being, good health and good financial status. Other factors identified by the study as predictors of good QOL were good health of the patient, patients' independence and more help in care giving. Factors perceived as negative predictors were concerns about the future, progression of the patient's illness and stress.

Despite the growing attention that QOL studies have received in recent years, some aspects should be discussed and investigated. First, none of the studies assessed many variables related to the symptoms so as to identify which symptoms are most influential on patient's QOL. Moreover, it is very important to identify differences in the influence of symptoms related to the stages of dementia.

Our aim in this study was to evaluate the influence of AD symptoms (mild and moderate dementia) on QOL of patients and their caregivers.

## Methods

### Participants

A cross-sectional study was conducted in a sample of AD patients and their caregivers. The study was conducted at the Neurology Outpatient Unit of the Hospital das Clínicas of the University of São Paulo School of Medicine, in São Paulo, Brazil. A total of 120 individuals, comprising 30 patients with mild AD, 30 patients with moderate AD and their caregivers/family members (n=60) participated in this study.

The group of patients (60) was composed of elderly people with a diagnosis of probable AD, according to the NINCDS-ADRDA criteria,^15^ having mild to moderate severity of dementia according to DSM-III-R criteria^16^ and based on scores on the Mini-Mental State Examination (MMSE).^17^ We used the Brazilian classification of MMSE scores according to educational level.^18^ Regarding the latter, scores had to fall between 10 and 25, both inclusive.

In order to participate in this study, patients had to be 60 years or older, have the presence of a family caregiver and not present serious impairment of language based on observation of understanding and expression of ideas at the time of the interview.

The caregivers group (n=60) comprised the patients' family caregivers. We considered as caregivers, family members who had a minimum of 24 hours weekly contact time with the patient in order to ensure reliability of collected information. All caregivers who answered the QOL-AD and the other instruments, were the patient’s main caregiver; no professional caregivers took part in the study.

Patients and caregivers were assessed between March 2002 and December 2005. The study was approved by the Ethics Committee of the Hospital das Clinicas of the University of São Paulo School of Medicine and written informed consent was obtained from all patients and from the caregivers recruited.

### Design and instruments

To assess QOL of patients and caregivers we used the QOL-AD proposed by Logsdon et al.,^3^ cross-culturally adapted and validated for the Portuguese language by Novelli et al.^19, 20^

The scale has three versions for the assessment of QOL: patients' reports (PQOL) and caregivers' reports (C-PQOL) on the patients' QOL, and a third version that focuses on QOL of the caregivers (CQOL). The scale also allows the combination of the self-report and the proxy report into a total score incorporating the patients’ and the caregivers/family versions, called composite version.^3,21^

For the evaluation of cognitive function we used the Mini-Mental State Examination (MMSE).^17,18^ We also applied the Physical and Instrumental-Self Maintenance scales (IADL and ADL)^22^ for functional performance evaluation, while the Neuropsychiatric Inventory (NPI)^23,24^ was used to assess behavioral disturbances. For mood evaluation we applied the Geriatric Depression Scale (GDS)^25,26^ and Cornell Scale for Depression in Dementia^27-29^ to the patients, and the Beck Depression Inventory to caregivers.^30,31^

### Procedures

Demographic clinical and QOL data from patients and caregivers were gathered by the first author of the study (MMPCN; occupational therapist). The following instruments were applied to the patients: socio-demographic information questionnaire, PQOL, MMSE, GDS and Cornell scales. Among the caregivers, the following instruments were applied: socio-demographic information questionnaire, C-PQOL, CQOL, IADL, ADL, NPI, Cornell scales and Beck Depression. The caregivers were also asked to confirm the patients’ socio-demographic information.

### Statistical analysis

A descriptive analysis (means and standard deviation) was undertaken for the socio-demographic variables of the sample.^32^

Kruskall- Wallis^32^ was employed to analyze age and education and the Chi-square^32^ to analyze gender, comparing the AD groups with mild and moderate dementia.

Pearson's correlation coefficient^32^ was employed for correlation of scores among QOL-AD and the other instruments. Linear regression (stepwise model) was performed to evaluate the influence of disease symptomatology on QOL both in mild and moderate dementia for patients and caregivers.

## Results

Socio-demographic characteristics of patients and caregivers are depicted in Table 1. There were no significant differences in relation to gender, age and years of schooling between AD patients. As expected, there were more female subjects in both groups of patients and caregivers and most of the caregivers, in both groups, were daughters or wives.

**Table 1:** Socio-demographic profile of patients and their respective caregivers/family members.

Socio-demographic characteristics	Mild dementia (n=30)	Moderate dementia (n=30)	p value (two tailed)
Age (patients)	75.5^a^±6.7^b^	76.2^a^±6.2^b^	p=0.70
Gender (patients)	Fem - 73.3 %	Fem - 66.7%	p=0.60
	Masc - 26.7%	Masc - 33.3%	
Educational level (patients)	6.5^a^±4.3^b^	6.0^a^±4.3^b^	p=0.68
Age (caregivers)	59.5^a^±15.4^b^	60.1^a^±14.5^b^	p=0.87
Gender (caregivers)	Fem - 80.0%	Fem - 66.7%	p=0.62
	Masc - 20.0%	Masc - 33.3%	
Educational level (caregivers)	10.0^a^±4.4^b^	9.1^a^±4.0^b^	p=0.44
Relationship	wife - 20.0%	wife - 30.0%	-
	sister - 20.0%	sister - 6.7%	
	daughter - 36.7%	daughter - 30.0%	
	husband - 16.7%	husband - 16.7%	
	son - 3.3%	son - 13.3%	
	granddaughter - 3.3%	grandson - 3.3%	

a Mean;

b standard deviations.

The scores obtained using the QOL-AD scale as well as with the other instruments applied to patients with mild and moderate dementia, and to their family caregivers are presented in Table 2.

**Table 2:** Mean and standard deviations of scores on instruments in mild and moderate AD.

Variables	Mild dementia (n=30)	Moderate dementia (n=30)	p value
PQOL^a^	36.8±5.2	36.1±5.3	0.63
C-PQOL^b^	33.3±5.0	28.3±6.0	0.00
CQOL^c^	38.6±6.4	35.2±5.5	0.04
Composite version^d^	35.6±4.4	33.52±4.6	0.18
Mini-Mental State Exam	20.2±2.6	14.0±2.8	0.00
Instrumental Activities of Daily Living (Lawton)^e^	16.5±4.9	23.3±3.4	0.00
Activities of Daily Living (Lawton)^e^	7.5±1.2	9.5±2.3	0.00
Neuropsychiatric Inventory	10.0±11.2	21.6±15.5	0.00
Geriatric Depression Scale (patient self-report)	8.1±5.3	8.6±6.0	0.76
Cornell scale for depression	5.4±4.9	8.9±4.8	0.03
Beck Depression	9.3±7.1	9.2±6.0	0.98

a PQOL (Patient reports);

b C-PQOL (caregiver reports on patient QOL);

c CQOL (Caregiver reports on own QOL);

d Composite version (comprising patient and family caregiver reports on patient quality of life;

e Physical and Instrumental Self-Maintenance Scale.

As expected, QOL scores on the three versions were lower in the moderate stage compared to mild dementia. No significant differences in patient reports were found. In caregiver reports about patients QOL and own QOL, we found significant differences when we compared mild and moderate dementia. The same reasoning applies to MMSE. On the order hand, our results showed an increase in total score for some variables such as mood, behavioral disturbances and performance on IADL and ADL, indicating a worsening condition when comparing mild to moderate dementia. There were statistically significant differences between the performance of the two groups on the major variables evaluated (p<0.05).

Table 3 shows the influence of the variables on QOL in mild dementia based on the PQOL, C-PQOL, CQOL and the composite version.

**Table 3:** Pearson's correlation between QOL-AD scale and other instruments in mild dementia.

Evaluations	PQOL^a^	C-PQOL^b^	Composite version^d^	CQOL^c^
Mini-Mental State Exam	0.04	0.03	0.05	0.13
Geriatric Depression Scale (patients self-report)	-0.76**	-0.48**	-0.78**	-0.14
Cornell Scale for depression	-0.49**	-0.68**	-0.63**	-0.20
Instrumental Activities of Daily Livinge	0.01	-0.52**	-0.02	-0.35*
Activities of Daily Living^e^	-0.21	-0.51**	-0.12	-0.02
Neuropsychiatric Inventory	-0.36*	-0.71**	-0.59**	-0.43*
Beck depression	-0.05	-0.27*	-0.19	-0.67**
PQOL^a^	-	0.41*	0.94**	0.06
C-PQOL^b^	0.41*	-	0.70**	0.26
CQOL^c^	0.06	0.26	0.15	-

* p<0.05;

** p<0.01.

a PQOL (Patient reports);

b C-PQOL (Caregiver reports on patient QOL);

c CQOL (Caregiver reports on own QOL);

d Composite version (comprising patient and family caregiver reports on patient quality of life;

e Physical and Instrumental Self-Maintenance Scale.

In mild dementia, we observed significant correlations between the PQOL version with depressive symptoms and behavioral disturbances. In the C-PQOL version, significant correlations were related to depressive symptoms, behavioral disturbances and IADL performance. The composite version was significantly correlated with depressive symptoms and behavioral disturbances. On the CQOL version, significant correlations were found for behavior disturbances, IADL performance and depressive symptoms reported by caregivers.

In the regression analyses, considering data from the mild dementia subgroup, the best QOL predictor on the PQOL version was depressive symptoms presented by patients (beta= –0.75 p=0.000). On the C-PQOL version, the best predictor was behavioral disturbances (beta= –0.62 p=0.000), while for the composite version, depressive symptoms reported by patients (beta= –0.78 p=0.000) and by the caregiver on the patient (beta= –0.34 p=0.008) proved best predictors. On the CQOL version, depressive symptoms reported by caregivers (beta= –0.67 p=0.000) were the best QOL predictor.

Table 4 shows the influence of different variables on QOL in moderate dementia based on the PQOL, C-PQOL, CQOL and the composite version.

**Table 4:** Pearson's correlation between QOL-AD scale and other instruments in moderate dementia.

Evaluations	PQOL^a^	C-PQOL^b^	Composite version^d^	CQOL^c^
Mini-Mental State Exam	0.04	0.27	0.15	0.22
Geriatric Depression Scale (patients self-report)	-0.45*	-0.30	-0.48**	-0.05
Cornell Scale for depression	-0.46**	-0.68**	-0.66**	0.27
Instrumental Activities of Daily Living^e^	-0.02	-0.41*	-0.19	-0.28
Activities of Daily Living^e^	-0.29	-0.49**	-0.43**	-0.38*
Neuropsychiatric Inventory	-0.31*	-0.67**	-0.53**	-0.23
Beck depression	0.02	-0.33*	-0.13	-0.54**
PQOL^a^	-	0.31	0.94**	0.21
C-PQOL^b^	0.31	-	0.70**	0.48**
CQOL^c^	0.21	0.48**	0.15	-

* p<0.05;

** p<0.01;

a PQOL (Patient reports);

b C-PQOL (Caregiver reports on patient QOL);

c CQOL (Caregiver reports on own QOL);

d Composite version (comprising patient and family caregiver reports on patient quality of life;

e Physical and Instrumental Self-Maintenance Scale.

In moderate dementia we observed significant correlations of the PQOL version with depressive symptoms and behavioral disturbances. Regarding the C-PQOL version, significant correlations emerged with depressive symptoms, IADL, ADL and with behavioral disturbances. On the composite version we observed significant correlations with depressive symptoms, behavioral disturbances and ADL performance. Finally, on the CQOL scale, significant correlations were related to depressive symptoms reported by caregivers about themselves and to ADL performance.

Regression analyses showed that the best predictor of PQOL scores in moderate dementia was depressive symptoms reported by caregivers about patients (beta= –0.48 p=0.009). Regarding the C-PQOL, the best QOL predictors were depressive symptoms reported by caregivers about the patients (beta= –0.68 p=0.000) and reported by caregivers about own depression (beta= –0.28 p=0.04). On the composite version, the best QOL predictor was depressive symptomatology reported by the caregivers about the patients (beta= –0.66 p=0.000), and ADL performance (beta= –0.56 p=0.000) and depressive symptoms reported by caregivers about own depression (beta= –0.41 p= 0.007) were the best QOL predictors in the CQOL version.

## Discussion

In the current study we examined the association between QOL evaluations and different clinical measures, including cognitive functions, IADL and ADL performance, behavioral disturbances and depressive symptoms in both patients and their caregivers. For these evaluations we used the three versions of the QOL-AD scale (PQOL, C-PQOL and CQOL) and the composite version (self-report and proxy report) in mild and moderate dementia.

AD symptoms affect not only patients’, but also caregivers / family members QOL. The evaluation of several variables associated with symptoms enabled us to identify the impact of the symptoms on QOL and the best predictor of QOL in mild and moderate dementia considering patients’ and caregivers' reports about patients’ condition and caregivers reports about themselves.

In the present investigation, we confirmed the influence of the symptoms on patients' QOL and their caregivers' quality of life.

In mild dementia, patients' QOL was associated with depressive symptoms reported by patients and caregivers about the patients, as well as behavioral disturbances and IADL performance. In relation to caregivers' version, QOL was influenced by the same variables.

The correlation with IADL performance was expected since these activities are affected in the early stages of dementia.^33,34^ These activities are complex and more cognitively demanding. Impaired performance makes patients face the limitations caused by the disease, with functional dependence.

In relation to depressive symptoms self-reported by patients, some studies^3,10^ had previously identified the influence of this variable on patients’ QOL. Depressive symptoms were associated with the reports by patients and caregivers about patients' QOL. Moreover, we also identified an association between depressive symptoms reported by caregivers (about themselves) and their own QOL. These symptoms affect the individuals' interest and motivation in various aspects of life, compromising their involvement in activities that make up part of everyday life, including pleasurable events. Logsdon et al.^3^ found an association between QOL and pleasurable events. Behavioral disturbances are common symptoms in all stages of the disease and our results showed that they affected QOL in both patients and caregivers, similar to findings of other studies.^3,10-12^ The most frequent behaviors in AD and dementia are apathy, depression, aberrant motor activity, anxiety, agitation/aggression, sleep alterations, delusions and hallucinations.^24,35^ The appropriate management of behavioral disturbances has been reported as one of the distressing factors for both patients and caregivers.

This study found no correlations between QOL measures and cognitive impairment, corroborating reports by other investigators.^3,10^ The absence of correlation does not imply that cognitive impairment does not affect QOL, but that other variables may have a greater influence, such as behavioral changes and depressive symptoms, as shown by the results. Moreover, it is important to note that anosognosia presented by AD patients may also explain this feature.

In moderate dementia, significant associations were found between patients' QOL and depressive symptoms reported by patients and their caregivers (about patients), behavioral disturbances and ADL performance. ADL performance is affected at this stage of the disease.^33,34^ Comparison with other studies can only be made considering patients’ self-reports, since no other studies have evaluated the influence of these variables on the proxy-report, composite version or the CQOL version of QOL.

We found differences in QOL on self-report and proxy-report. These differences could be explained by the presence of anosognosia in patients, hindering the perception of the limitations, difficulties and deficits. Another important aspect is the fact that the caregivers use their own beliefs, values and emotional state to assess the patients' QOL. Despite these discrepancies, both reports were correlated with QOL evaluation where the combined methods to evaluate QOL proved the best method.

Regression analysis allowed identification of the best QOL predictor in all reports and across different levels of the disease. Our results indicated that the symptoms of the disease affected QOL in AD.

The main finding was the identification of the best QOL predictor based on both patients' and caregivers' QOL. This is the first study evaluating patients' and caregivers' QOL within the same sample, exploring several variables associated with the disease. We believe this study provides important information about the impact of these variables' on QOL in AD. This study also provides insights on the impact of the different symptoms allowing the proposal of pharmacological and non-pharmacological interventions to improve QOL of both patients and caregivers.

Nevertheless, the present study has some important limitations that need to be addressed. The study design was cross-sectional and the proposed hypothesis could be more appropriately tested in longitudinal investigations. In addition, the studied sample was small and focused only on mild and moderate dementia. Hence, results may not be generalized to other populations.

Further studies involving a larger sample need to be performed in order to evaluate the influence of other variables on QOL, including cultural and socio-demographic aspects, network support and the impact of the environment.

Despite these limitations, this study supports the notion that QOL of patients and their caregivers is influenced by specific disease symptoms.
